# A case of nodular lymphocyte predominant Hodgkin lymphoma with unexpected EBV-latency type

**DOI:** 10.1007/s00277-020-04174-4

**Published:** 2020-07-19

**Authors:** Mathias T. Rosenfeldt, Elena M. Hartmann, Corinna Leng, Andreas Rosenwald, Ioannis Anagnostopoulos

**Affiliations:** 1 Institute of Pathology, University of Wuerzburg, Josef-Schneider Str. 2, 97080 Wuerzburg, Germany; 2 Comprehensive Cancer Center Mainfranken, Josef-Schneider Str. 6, 97080 Wuerzburg, Germany; 3 Division of Hematology, Oncology, and Tumor Immunology, Campus Benjamin Franklin, Charité-Universitätsmedizin Berlin, Hindenburgdamm 30, 12203 Berlin, Germany

Dear Editor,

Hodgkin lymphoma (HL) comprises two major entities: classic Hodgkin lymphoma (CHL) in > 90% of cases and nodular lymphocyte predominant Hodgkin lymphoma (NLPHL) in the remaining 5–10%. Both are of B cell origin, but only NLPHL generally retains the B cell programme [1–3]. Only a minority (4–5%) of NLPHL are associated with Epstein-Barr virus (EBV, human herpesvirus 4 (HHV-4)), whereas in CHL, EBV can be found in up to 50% of cases [1–3]. EBV infection affects > 90% of adults worldwide. Primary infection is mostly asymptomatic but leads to a persistent latent infection [4]. EBV dormancy is characterised by specific latency programmes with differential expression of viral non-coding RNAs (e.g. EBV-encoded RNAs, EBERs), latent membrane proteins (LMPs) and EBV-nuclear antigens (EBNAs). EBERs are found in all latency states, whereas LMPs and EBNAs are variably expressed [5, 6]. Latency type II with expression of LMP1 and positive in situ hybridization (ISH) for EBERs is nearly ubiquitous in EBV-associated CHL. The latency pattern for EBV-positive NLPHL is not precisely established [1, 2, 6, 7].

**Fig. 1:**
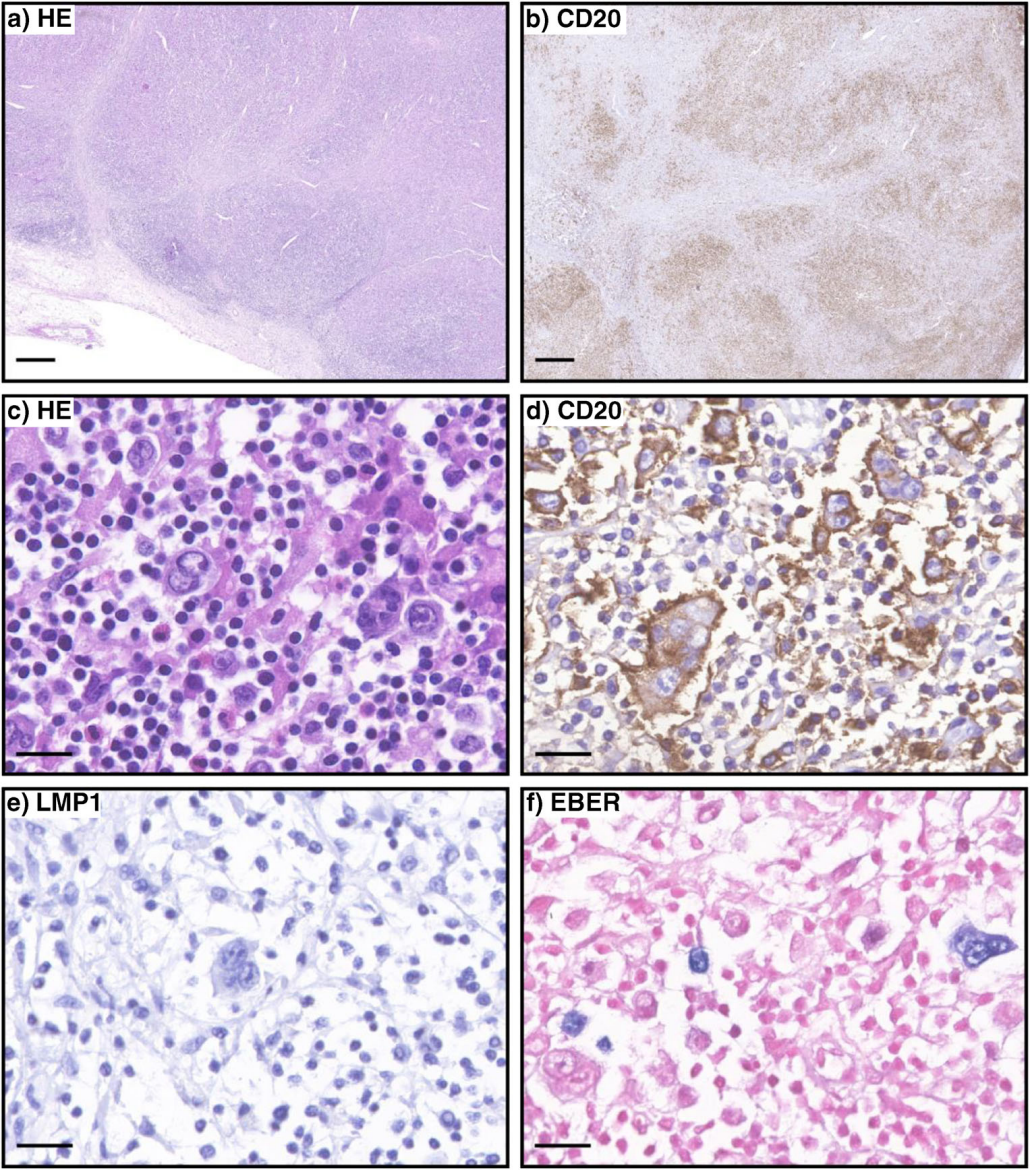
**a**–**f** Representative images stained as indicated. Scale bars in the top two panels represent 500 μm, whereas in the other panels, they represent 20 μm

We present a case of NLPHL with an unusual EBV latency type. We received a formalin fixed paraffin-embedded cervical lymph node of a 60-year-old female patient of Iranian descent. She presented with B-symptoms, cervical, abdominal and inguinal lymphadenopathy and splenomegaly. The past medical history and laboratory parameters were largely unremarkable. Lymph node architecture was effaced by a nodular infiltrate composed predominantly of small lymphocytes, histiocytes, sparse eosinophils and intermingled larger atypical cells, some with features of Hodgkin- and Reed-Sternberg cells, and others of LP cells (Fig. 1a, b, c). Morphology prompted a differential diagnosis of nodular sclerosis CHL vs the rare case of NLPHL with occasional eosinophils. Atypical cells co-expressed CD20 (Fig. 1d), CD79a, CD75, OCT-2, BOB.1 and BCL6, while CD30 was detected only in single, non-neoplastic bystander cells. CD21 highlighted the presence of follicular dendritic cell meshworks with embedded tumour cells. Immunohistochemistry for PD1 identified rosettes of PD1-positve T cells that surrounded LP-cells. Clonality analysis employing the BIOMED-2 protocol ruled out the presence of a clonal T cell population. These findings prompted a diagnosis of NLPHL and ruled out all differential diagnoses. Repeated immunostaining for LMP-1 was negative (Fig. 1e). Unexpectedly, a majority of tumour cells harboured EBER transcripts, albeit a minority remained negative (Fig. 1f), indicating a latency gene expression pattern other than type II, potentially type I. Moreover, sparse EBER-ISH-positive small lymphoid cells of the microenvironment were detectable. The literature covering EBV in NLPHL is limited [2, 8, 9]. Positive EBER-ISH and LMP1 expression have been reported, as well as positive EBER-ISH but negativity for LMP1 and even a case with probably artificially negative EBER-ISH and positivity for LMP1 [2, 8, 9]. Nonetheless, the available literature implies that unlike CHL, NLPHL is not unequivocally associated with a certain EBV latency gene expression pattern. We postulate that NLPHL demonstrates a broad spectrum of EBV latency patterns and that it is worthwhile to examine these in order to gain further insights into the complexity of this disease.
